# Retraction: Comprehensive Comparison of Three Different Immunosuppressive Regimens for Liver Transplant Patients with Hepatocellular Carcinoma: Steroid-Free Immunosuppression, Induction Immunosuppression and Standard Immunosuppression

**DOI:** 10.1371/journal.pone.0222507

**Published:** 2019-09-10

**Authors:** The PLOS ONE Editors

Concerns have been raised that the transplants performed in the local context at the time of procedures reported in this article [1] may have involved organs/tissues procured from prisoners [2].

The Ethics Statement of [1], declares, “None of the transplant donors were from a vulnerable population and all donors gave their consent freely.” However, the authors did not provide ethics approval documentation or consent forms when requested by the journal to support their claims, confirm that the study had institutional ethics approval, and clarify whether organs had been procured from prisoners. Details as to the donor sources and methods of obtaining informed consent from donors were not reported in [1], and the authors did not respond to the journal’s queries to clarify these issues or the cause(s) of donor death. International ethics standards call for transparency in organ donor and transplantation programs and clear informed consent procedures including considerations to ensure that donors are not subject to coercion.

In addition, the authors did not provide the primary data underlying their article and as such, did not comply with the PLOS Data Availability Policy.

Owing to the lack of documentation to demonstrate this study had prospective ethical approval, insufficient reporting, unresolved concerns around the source of transplanted organs and whether they included organs from prisoners, and in compliance with international ethical standards for organ/tissue donation and transplantation, the *PLOS ONE* Editors retract this article.

The authors did not respond to the retraction decision.
